# Contact Depth Affects Most of the Other Hitting Metrics We Analyze

**DOI:** 10.3390/sports14080330

**Published:** 2026-08-03

**Authors:** Kevin A. Giordano, Ian Jump, Benjamin Lerch, Gretchen D. Oliver

**Affiliations:** 1 Department of Physical Therapy, Louisiana State University Health Sciences Center, 1900 Gravier St., New Orleans, LA 70112, USA; 2 School of Kinesiology, Auburn University, 301 Wire Rd., Auburn, AL 36803, USA; izj0017@auburn.edu (I.J.); bgl0015@auburn.edu (B.L.); gdo0001@auburn.edu (G.D.O.)

**Keywords:** baseball, in-game, motion capture, swing metrics

## Abstract

Biomechanical theory and coaching heuristics suggest that contact depth meaningfully constrains swing mechanics and batted-ball outcomes. Our purpose was to examine the relationship between contact depth and key hitting metrics during live collegiate competition. Markerless motion capture and ball tracking data were collected during live NCAA Division I games from 2779 batted balls from 239 hitters. Outcomes included contact depth, exit velocity, maximum swing speed, swing speed at contact, and vertical and horizontal attack angles. Regressions assessed relationships with velocity and swing speed variables, while partial least squares regression evaluated attack angles. The mean contact depth was 20.1 ± 7.2 inches in front of the hitter’s center of mass. Contact depth weakly explained exit velocity (r^2^ = 0.04, *p* < 0.001) but significantly predicted maximum swing speed (r^2^ = 0.20 *p* < 0.001). No meaningful relationship was observed with swing speed at ball contact (r^2^ = 0.01, *p* > 0.01). Contact depth showed a strong association with the combined structure of vertical and horizontal attack angles, with the primary PLS component explaining 80.0% of the contact depth variance. Contact depth appeared to influence swing mechanics and attack angles and was associated with peak, but not contact, bat speed. These findings highlight contact depth as a critical covariate for interpreting swing metrics and designing biomechanical assessments, analytic models, and on-field interventions.

## 1. Introduction

Over the past decade, baseball analytics has undergone a rapid evolution driven by advances in tracking technology and the availability of detailed performance data. Metrics such as exit velocity, launch angle, and spray angle are now routinely used to evaluate hitters, forecast future performance, and inform pitching strategy. At the professional level, these variables underpin widely used constructs such as expected weighted on-base average (xwOBA) and are increasingly adopted at the collegiate level as tracking infrastructure expands. For pitchers and analysts, understanding the mechanics behind how hitters generate these outcomes is critical for developing tailored approach strategies. Additionally, for hitters, monitoring these metrics supports progress tracking and player development initiatives.

Despite these advancements, several aspects of the batting process that plausibly influence batted-ball outcomes remain underexplored in public and academic domains. One such factor is contact depth, the longitudinal location along the hitter–pitcher axis at which ball contact occurs relative to the hitter’s body. From a biomechanical perspective, contact depth is theoretically linked to swing plane orientation, bat speed generation, and the resulting direction and quality of the batted ball. In a laboratory study, Morishita et al. found contact depth to be a leading predictor of bat velocity [1]. The curvilinear path of the swing would result in contact depth farther in front of the plate displaying an increased attack angle and a more pull-side-oriented horizontal attack angle. There is also evidence indicating a kinematics change based on the contact point in the strike zone, which would plausibly influence attack angles [2]. Further, as the axis of rotation of the bat travels distally from the body during the swing, the bat head would have greater linear velocity. From a practicality standpoint, the swing is likely most powerful around a given radius, so the hitter will alter their swing mechanics to hit the ball where the pitch intersects this swing radius [3,4]. Practitioners and coaches frequently reference concepts such as “catching the ball out front” or “letting the ball travel”, yet these ideas are rarely quantified in large-scale datasets and are largely absent from the peer-reviewed literature.

The limited experimental work that does exist suggests that contact location may influence swing mechanics and performance outcomes. For example, Misaki et al. demonstrated that contact made closer to the ground on a batting tee was associated with reduced contact precision and lower vertical attack angles, implying a mechanical constraint imposed by the contact location [5]. Similarly, Nakashima et al. showed that variations in bat path influence the timing error tolerance of the swings (how mistimed the swing can be and still result in ball contact), indirectly implicating contact depth as a critical determinant of successful ball contact [6]. For both tee and machine batting, horizontal and vertical contact location both influenced contact depth [3,4]. However, these studies did not study directly quantified contact depth during live pitching conditions, nor did they examine its relationship with modern bat path metrics or exit velocity.

Increased data availability has improved the rigor of hitting analyses using in-game data [7,8,9]. However, contact depth is typically not available in most commercial datasets, which limits its application in advanced baseball analytics. As a result, analysts either infer its effects indirectly through launch angle, spray angle, or batted-ball distributions, or they interpret other bat path metrics without considering contact depth. This omission is notable, as contact depth may explain observed variability in these commonly reported metrics.

Therefore, the purpose of this study was to explore the relationship between contact depth and several key hitting variables: exit velocity, maximum swing speed, swing speed at ball contact, vertical attack angle, and horizontal attack angle. Using in-stadium markerless motion capture and radar-based ball tracking data collected during a live National College Athletic Association (NCAA) Division I competition, this investigation aimed to provide one of the first large-scale, in-game analyses of contact depth and its biomechanical and performance correlates. We hypothesized that a contact depth closer to the catcher would be associated with decreased horizontal and vertical attack angles, decreased peak and contact bat speed, and decreased exit velocity.

## 2. Materials and Methods

### 2.1. Participants and Ethical Approval

The study received exempt approval from our Institutional Review Board (protocol 23-218 EX 2304). Participants were included if they were playing on an NCAA Division I roster that competed at the home institution’s baseball field, and had an at-bat where they put the ball in play (not a walk, strikeout, or hit by pitch).

### 2.2. Research Design

The current study used an observational, cross-sectional design, consistent with previous research in the field of sport science [10]. The study consisted of examining the association between contact depth and swing performance metrics in an in-game setting without experimental manipulation.

### 2.3. Experimental Design

Kinematic, bat, and ball tracking data were collected from 239 NCAA Division I hitters over the course of the 2024 and 2025 collegiate baseball seasons. Data were obtained during live game competitions using a combination of eight fixed, in-stadium KinaTrax markerless motion capture cameras (300 Hz) and TrackMan™ radar technology. While validation for markerless technologies in game is not feasible to perform, in-stadium validation has indicated good validity for many kinematic measurements with markered motion capture, although it has only been performed in pitching [11]. Cameras were calibrated at the start of each season and monitored by the vendor for data quality for any needed recalibrations following camera maintenance. Calibration was performed using a 3 × 3’ (91.44 cm) fixed grid followed by a standardized checkerboard being waved in front of each camera at the home plate. Drift corrections were performed after every game. While Kinatrax has not released academic literature on its calibration, similar techniques have been described [11,12]. A total of 2779 batted balls put into the field of play were included in the analysis. Bunts and check swings were excluded due to their fundamentally different mechanical intent and execution. Only swings that resulted in fair or foul balls in play were retained, ensuring that all observations reflected meaningful ball contact under game conditions.

**Figure 1:**
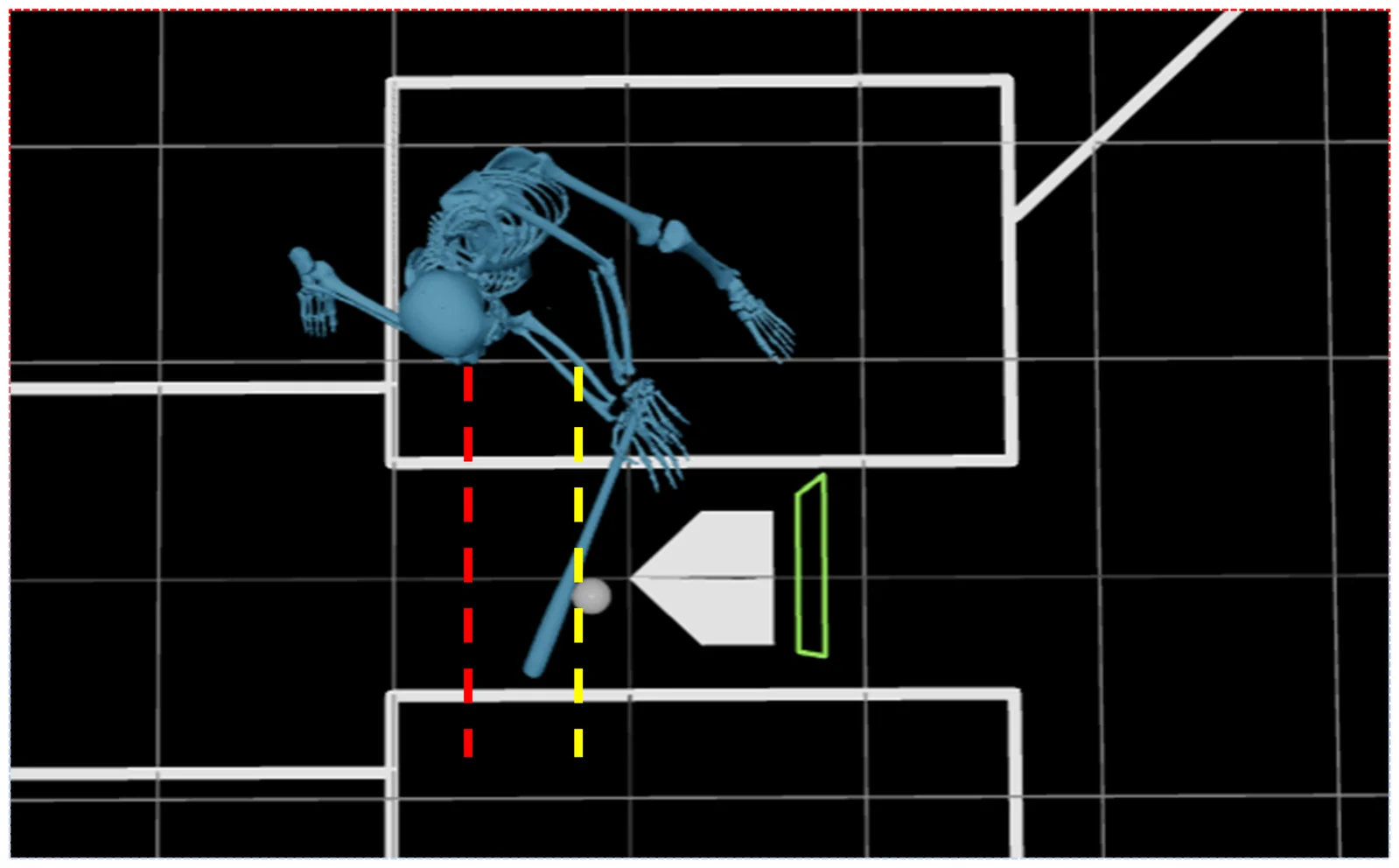
Contact depth visual. Yellow line represents ball contact, red line represents center of mass location, and distance between the two of them represents contact depth.

The contact depth was operationally defined as the linear distance, in inches, between the hitter’s center of mass (COM) and the location of ball contact along the axis from the home plate toward the pitcher’s mound (Figure 1). The COM was calculated from anthropometric normative data derived from the height and weight for each segment, and then a centroid was taken from the summation. Positive values indicated contact occurring in front of the hitter’s COM, whereas negative values indicated contact occurring behind the COM (deeper in the hitting zone). The hitter’s COM was estimated using the full-body kinematic model generated by the KinaTrax system. The ball contact location was derived from synchronized bat and ball tracking data, allowing for the precise spatial determination of the collision point in three-dimensional space. The contact depth was calculated for each batted ball as a scalar projection along the home plate-to–mound vector (the distance of the ball along the first–third base axis was ignored).

The variables of interest were the: (1) exit velocity in MPH, (2) maximum swing speed in MPH (80% of the distance from the knob to the cap), (3) swing speed at ball contact (80% of the distance from the knob to the cap) in MPH, (4) vertical attack angle in degrees, and (5) horizontal attack angle in degrees.

### 2.4. Statistical Analysis

Descriptive statistics were calculated for contact depth across all batted balls. To assess the relationship between contact depth and exit velocity, a quadratic linear regression model was employed. This approach was selected a priori to allow for potential non-linear relationships, as both excessively deep and excessively forward contact may plausibly reduce energy transfer efficiency. Simple linear regressions were used to evaluate the influence of contact depth on maximum swing speed and swing speed at ball contact. We were comfortable using linear regressions for these variables for predictive simplicity because we hypothesized that the number of ball contacts occurring far in front of the plate enough to substantially alter swing speed would be minimal. Model assumptions were visually assessed via residuals. Vertical and horizontal attack angles were expected to exhibit substantial collinearity, as both are derived from the same bat velocity vector at contact. Therefore, partial least squares (PLS) regression was used to examine the relationship between contact depth and the combined structure of vertical and horizontal attack angles. The proportion of variance explained by each PLS component was calculated to assess the strength of association. Of note, we did not use any form of a multilevel model in these analyses because home team hitters from the host institution contributed most of the batted balls and visiting hitters had as few as one ball in play, which made the incorporation of random effects into multilevel modeling infeasible. Our author group believes that the relationship that contact depth has with our variables of interest is consistent between hitters, which was visually assessed, and therefore accepted the partial pooling limitation—the greater sample size from visiting hitters was worth the model limitation. All statistical analyses were conducted using R (Version 4.4.1) [13]. Data were imported using the readr package (version 2.2.0) [14], visualized using ggplot2 (version 4.0.2) [15], and analyzed using linear regression models implemented in base R (stats package) [13]. Partial least squares (PLS) regression was performed using the pls package (version 2.9.0) [16]. Effect sizes were categorized based on work by Brydges [17]. Therefore, we interpreted variance that accounted for >0.014 and <0.04 as small, >0.04 and <0.10 as medium, and >0.10 as large. However, we encourage the reader to interpret effect sizes for their numerical influence on dependent variables, rather than arbitrary cutoff thresholds. Statistical significance was set a priori at *p* < 0.05 and a Benjamini–Hochburg correction was used to account for familywise error [18].

## 3. Results

**Figure 2:**
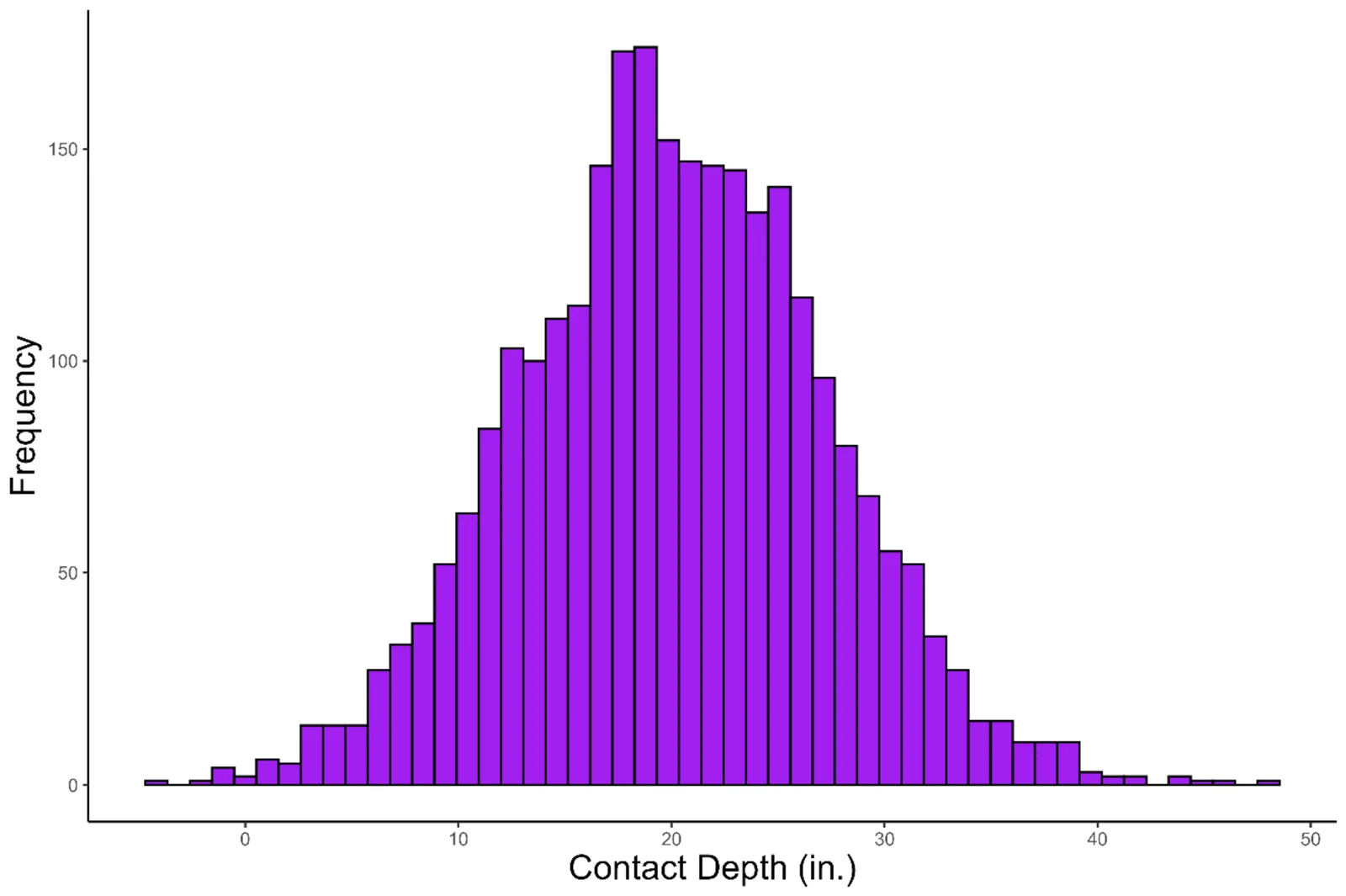
Histogram of where ball contact occurs compared to body’s center of mass in the direction from home plate to the pitcher’s mound.

The mean contact depth across all batted balls was 20.1 ± 7.2 inches in front of the hitter’s center of mass (Figure 2). The observed range of contact depth spanned from −4.3 inches (deep contact) to 47.9 inches (far out in front), indicating substantial variability in where hitters made contact relative to their bodies during live competition.

**Figure 3:**
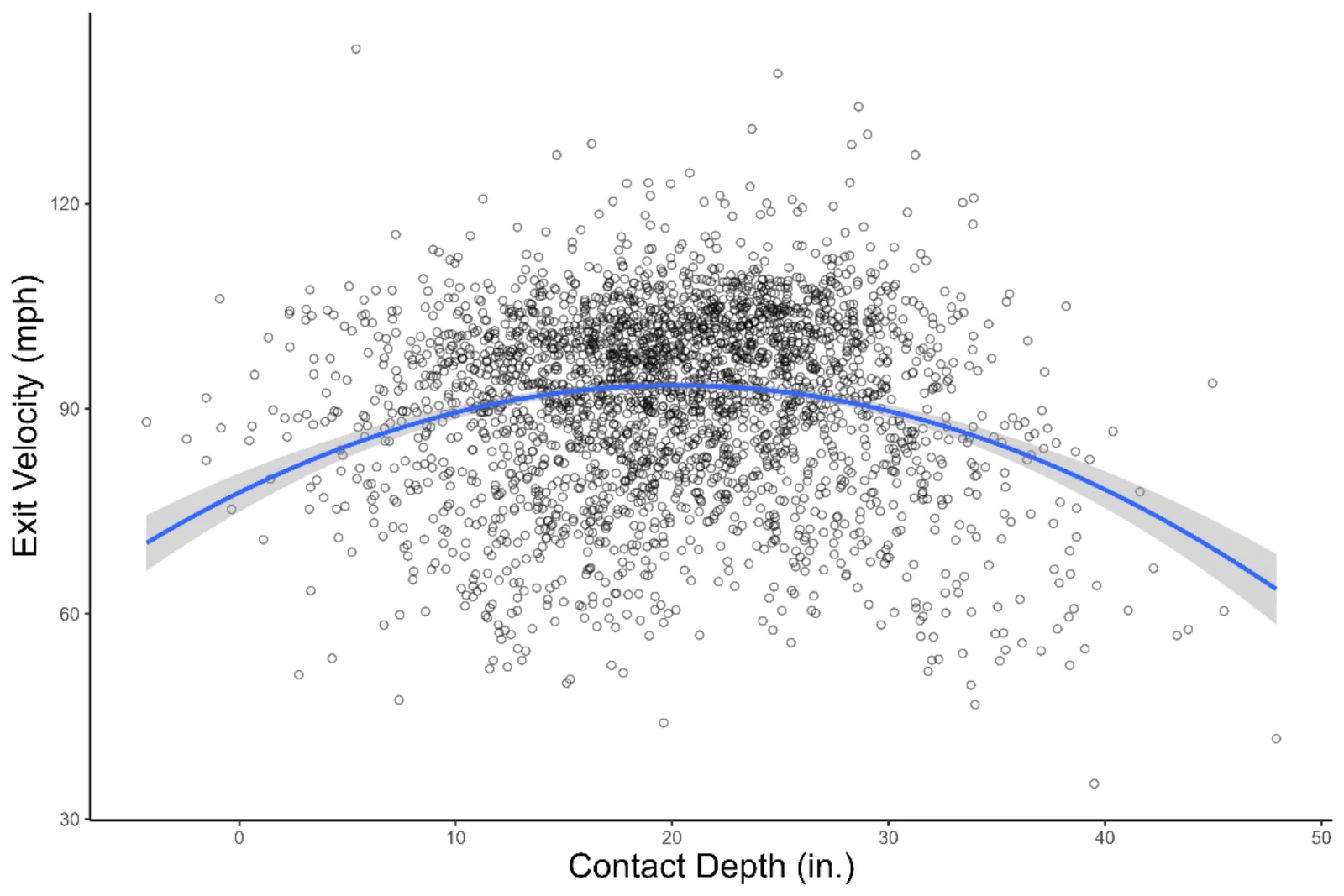
Contact depth and exit velocity. Positive depth values indicate toward the pitcher.

**Figure 4:**
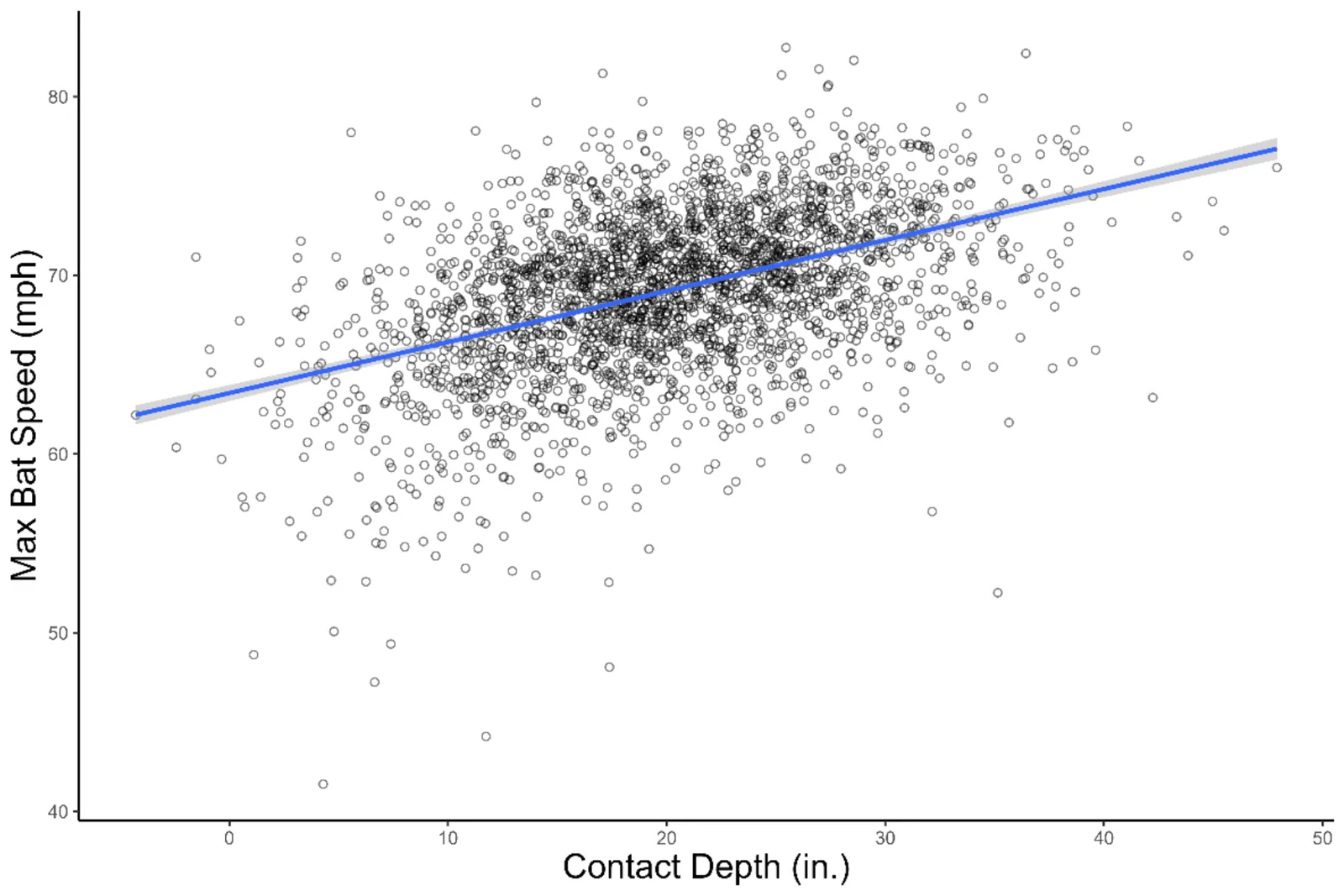
Contact depth and maximum bat speed during the swing. Positive depth values indicate toward the pitcher.

**Figure 5:**
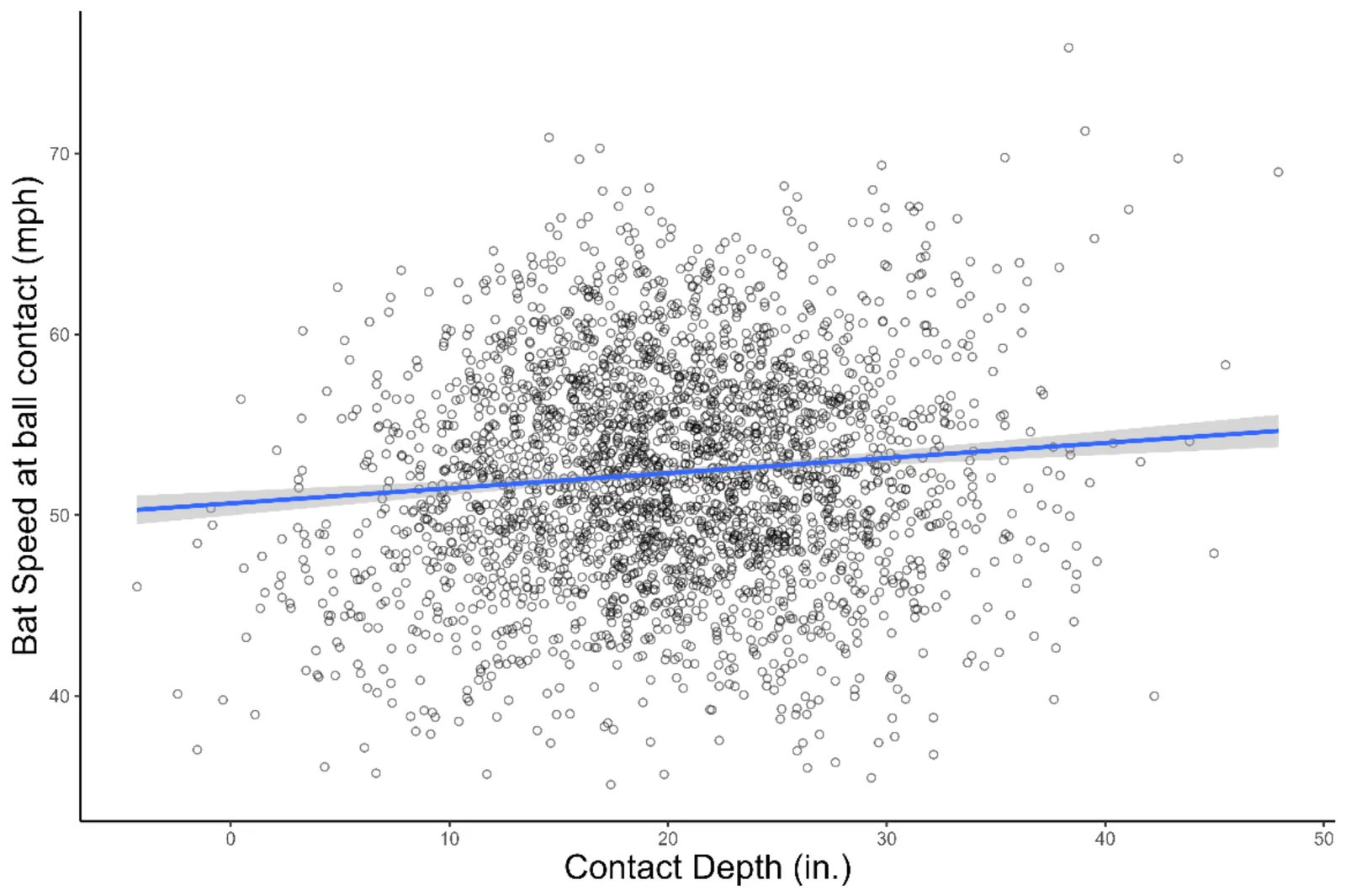
Contact depth and bat speed at ball contact. Positive depth values indicate toward the pitcher.

**Figure 6:**
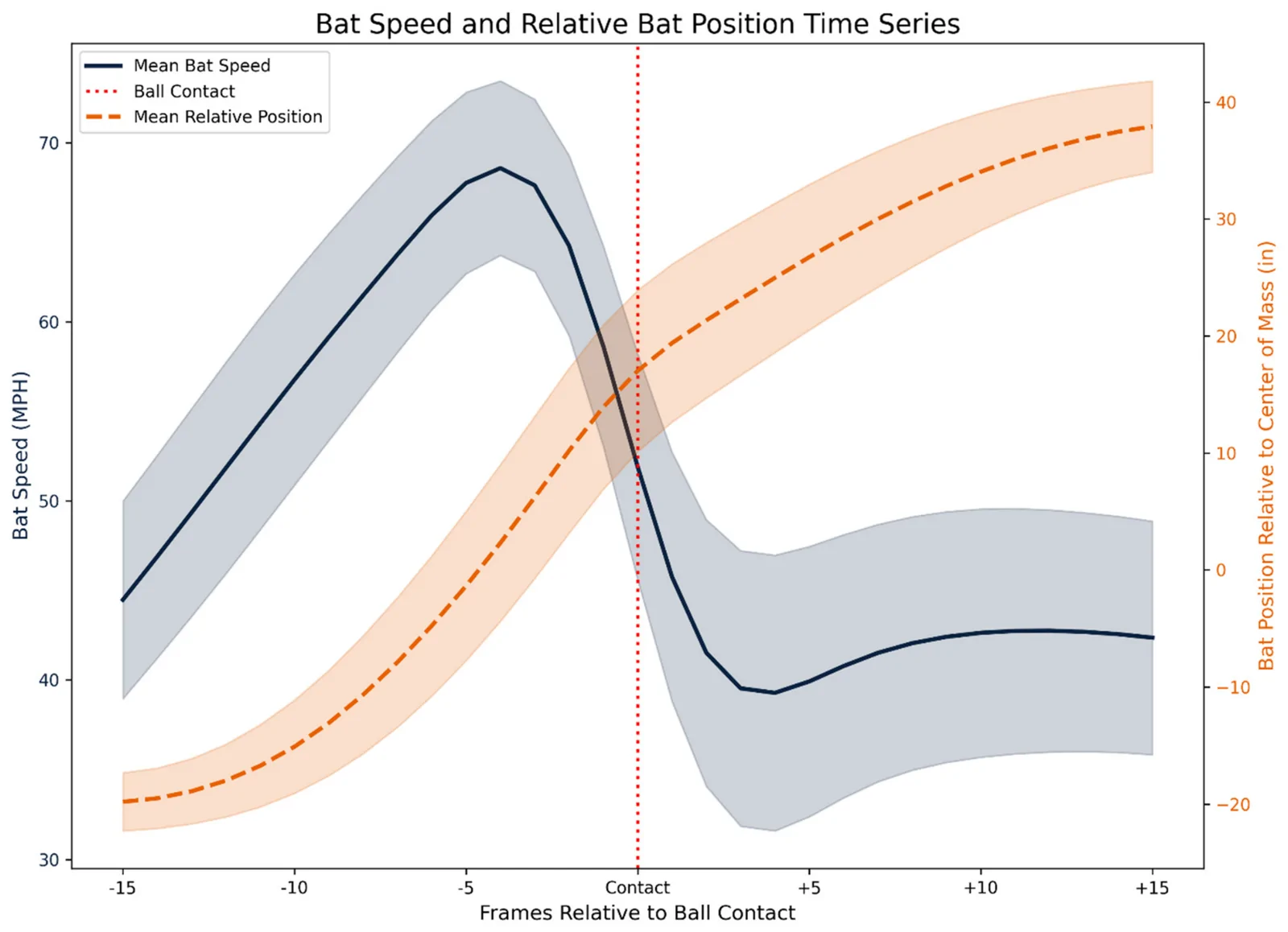
Bat Speed and Bat Position. Contact depth occurred, on average, 20.1 inches in front of the hitter’s COM. Plot is trimmed to 15 frames before and after ball contact to only track forward bat progression. Positive depth values indicate toward the pitcher. Note: Because this plot shows the location of the centroid of the bat, not the contact point from the centroid of the ball, the visual contact point on this plot appears slightly lower than the contact depth reported elsewhere in this paper.

Quadratic regression analysis revealed that contact depth weakly explained the variance in exit velocity (r^2^ = 0.04, *p* < 0.001). Although the overall model was statistically significant, the inspection of residuals demonstrated an asymmetric distribution, with positive residuals clustering above the mean exit velocity (Figure 3). Contact depth significantly predicted maximum swing speed (β = 0.29, r^2^ = 0.20, *p* < 0.001), indicating that swings associated with more forward contact tended to achieve greater peak bat speeds (Figure 4). In contrast, contact depth had no meaningful influence on swing speed at ball contact (β = 0.08, r^2^ = 0.01, *p* > 0.01) (Figure 5). Partial least squares regression confirmed strong covariance between vertical and horizontal attack angles. The first PLS component explained 68.7% of the shared variance between these two angles. Vertical and horizontal attack angle had loadings of 0.65 and 0.77 respectively, and weights of 0.56 and 0.83 respectively on the first PLS component. Importantly, this first component also explained 80.0% of the variance in contact depth, indicating a very strong relationship between contact depth and the combined structure of attack angles. To contextualize these data, a summary plot of the time series for both bat speed and bat position is provided (Figure 6).

## 4. Discussion

Contact depth is not a publicly available metric in large baseball datasets; however, many variables that are analyzed are influenced by contact depth. Analysts and coaches need to be aware of the influence of contact depth when using variables such as attack angle and bat speed for player evaluation and coaching intervention. The purpose of this study was to explore the relationship of contact depth with several key hitting variables commonly tracked as performance metrics or used for intervention purposes. We determined that contact depth influences exit velocity, maximum bat speed, and horizontal and vertical attack angles, but is a much weaker predictor of bat speed at ball contact. The typical profile for bat speed results from the distally progressing axis of rotation of the bat in the kinetic chain. Therefore, a notable, localized spike in bat speed occurs slightly in front of the hitter’s COM when contact is anticipated (Figure 6). Although hitters modify their swings to achieve different contact locations, we hypothesize that the variability in contact depth (Figure 2) exceeds the typical swing variability for different pitch locations [3,6], which may explain the substantial impact of contact depth on swing metrics observed in this study. While exit velocity was only weakly associated with contact depth (Figure 3), there was an abnormal cluster of residuals around the average contact depth. This pattern suggests that while contact depth alone has limited explanatory power in a traditional organized least squares analysis, certain contact depths may permit disproportionately large exit velocities under favorable swing conditions. A more refined analysis, such as cluster analysis to identify optimal contact depth ranges, could provide better insight. Additionally, our analysis did not incorporate ball flight metrics; refining the analysis by limiting spray and launch angle ranges could intensify the clustering of high exit velocities within specific contact depths. Combining ball flight data with exit velocity metrics could further identify optimal contact depths for maximizing exit velocity.

Our most noteworthy finding of this research is the observed relationship between contact depth and maximum bat speed coupled with the weak relationship between contact depth and bat speed at ball contact. Bat speed has gained tremendous popularity since Statcast began releasing bat speed data a few years ago [19]. On average, there is a clear positive relationship between a player’s average bat speed and meaningful offensive statistics [19]. Publicly reported bat speed metrics are reported at contact (or when contact would have occurred on a swing and miss). This dissociation suggests that while hitters may generate greater peak speeds when contacting the ball farther in front, the bat speed at the moment of collision is relatively conserved across contact depths at a population level. Whether hitters can hit the ball farther out in front to increase the bat speed at contact as a within-hitter adjustment still needs to be analyzed with a larger sample than the present study. For biomechanists conducting laboratory assessments, these findings are important for generalizability to a game setting. In a lab setting, off a tee or typical pitching machine, hitters can plan their contact depth without the reaction challenges of an in-game setting. Therefore, when positioning a tee, or allowing a hitter to preselect a spray angle to the best of their ability, contact depth may influence lab-measured bat speed while not being indicative of in-game bat speed at ball contact. The relationship of when the peak bat speed occurs relative to contact remains an area for future study.

Another important finding was that contact depth had a strong influence on both horizontal and vertical attack angles. Although this seems intuitive from the parabolic shape of the swing, many attack plans or swing interventions use vertical attack angle at contact as a metric representative of their swing. While there is a very likely signal to the discrete metric, controlling for contact depth is likely to reduce noise and residual variance in models analyzing swing path. We should note that while contact depth was clearly a covariate in attack angles, the causal arrow may be bidirectional as hitters purposefully allow the ball to travel deeper into the zone to hit the ball to the opposite field along with hitters being beat by a fastball/out in front of an off-speed pitch. These findings demonstrate that contact depth is tightly coupled with the orientation of the bat at contact, reinforcing the notion that where the hitter contacts the ball substantially constrains the resulting bat path and batted-ball direction. Only looking at swing angles without contextualizing contact depth is problematic.

For analysts/sport scientists, these insights carry several implications. First, contact depth should be incorporated as a covariate in models analyzing swing biomechanics. Second, when creating attack plans for pitchers, understanding contact depth and attack angles, rather than only using the discrete vertical attack angle, provides a more comprehensive picture of swing shape. For example, a hitter that hits to the opposite field may often have a much lower vertical attack angle, which may indicate vulnerability to low breaking balls. However, if the hitter only has a shallow attack angle due to a deeper contact point, their swing path may not be flatter than the average hitter, resulting in a flawed plan. Last, analysts should continue to scrutinize the concept of an “optimal contact depth” zone. This can be optimized by an individual hitter’s biomechanics and how this corresponds to proper swing decisions based on the hitter’s swing profile in that optimal contact depth zone.

For coaches and player development staff, they should be aware that looking at an attack angle may not provide a complete picture of the hitter’s swing. Coaches more familiar with biomechanics and swing metrics should blend contact point data with swing profile data. Coaches with less biomechanics training should blend simple swing profile data with their knowledge of a hitter’s tendencies/hitting approach to contextualize why a hitter may be succeeding or struggling with certain pitch types. Last, when intervening on a hitter’s swing to improve contact rates or batted-ball profiles, these data suggest two primary routes: (1) intervening on a hitter’s swing biomechanics to alter the swing path, or (2) drilling to change a hitter’s typical contact point, which will change their recorded swing metrics without altering swing biomechanics. These conclusions align with previous research, indicating that the contact point will alter the swing plane and biomechanics [4]. These findings also contextualize the swing path and margin for error in making contact with a moving pitcher, rather than a stationary ball [3,6].

This study should be interpreted with several limitations. First, as previously discussed, we did not account for the nested data structure because of the small cohort of home team players with many repeated measures, but the larger sample of hitters with very few balls batted into playwould have made a multilevel model difficult to interpret. We believe that population-level conclusions may be drawn, but this does limit our ability to interpret within-hitter adjustments, which we hypothesize may explain some of the lack of relationship between contact depth and bat speed at ball contact. Second, for simplicity and interpretability by coaches and players, we kept this analysis to contact depth, rather than the three-dimensional location of the contact point. There is likely a continuum of biomechanical compensations that occur in each cardinal plane that are driven by the distance the contact point is away from the hitter’s body COM, rather than just depth. Last, we remind the reader that motion technology is quickly improving, but there is measurement error in motion capture data, introducing noise into our models. Further, with KinaTrax being proprietary software, exact definitions and parameters for all variables are not entirely known.

## 5. Conclusions

Contact depth is an important biomechanical variable associated with several key swing characteristics during live NCAA Division I baseball competitions. More forward contact was associated with greater maximum bat speed and substantial changes in the combined structure of vertical and horizontal attack angles, whereas bat speed at ball contact was largely unaffected. Although contact depth explained only a small proportion of the variance in exit velocity, the observed relationships suggest that contact depth meaningfully influences how swing mechanics are expressed and, consequently, how commonly reported hitting metrics should be interpreted. These findings support incorporating contact depth into biomechanical analyses, player evaluation, and swing intervention strategies to provide greater context for bat path metrics and improve the interpretation of hitter performance during game play.

## Data Availability

Due to proprietary collection sources, data are not publicly available from this study. However, analysis source code is available upon request of the corresponding author.
